# Vitamin B_12_ and folate intake and biomarker status among omnivore and vegetarian UK-dwelling women of childbearing age: pooled results from the National diet and nutrition surveys (2008–2019)

**DOI:** 10.1007/s00394-026-04117-1

**Published:** 2026-10-13

**Authors:** Ali Niklewicz, Luciana Hannibal, Kourosh R. Ahmadi

**Affiliations:** 1 Department of Nutritional Sciences, School of Biosciences and Medicine, University of Surrey, Guildford, GU2 7XH UK; 2 Laboratory of Clinical Biochemistry and Metabolism, Department of General Paediatrics, Adolescent Medicine and Neonatology, Faculty of Medicine, Medical Centre, CIBSS-Centre for Integrative Biological Signalling Studies, University of Freiburg, 79106 Freiburg, Germany

**Keywords:** Plant-based diets, Vegetarian, Vitamin B_12_, Women of childbearing age

## Abstract

**Purpose:**

Vitamin B_12_ is crucial for neurological function and maternal health, particularly during pregnancy and lactation. Women of childbearing age (WCBA) following unfortified plant-based diets are at increased risk of B_12_ deficiency, which can adversely affect both maternal and infant health. This study analysed NDNS data (2008–2019) to: (1) compare vitamin B_12_ intake and status, and folate intake and status, between omnivores and vegetarians; (2) examine the impact of supplementation; (3) explore predictors of B_12_ biomarkers, including diet, supplementation, and age, and (4) assess the prevalence of vitamin B_12_ insufficiency and deficiency in WCBA, per diet group.

**Methods:**

Data from 2,299 WCBA (2,204 omnivores and 89 vegetarians) in the National Diet and Nutrition Survey (2008–2019) were analysed. Survey-weighted means and 95% confidence intervals (CI) were calculated for nutrient intakes and biomarkers, and t-tests compared omnivores and vegetarians. Vitamin B_12_ and folate intakes were assessed from food and supplements.

**Results:**

Mean dietary vitamin B_12_ intake was significantly higher in omnivores (4.3 µg/day; 95% CI: 4.2–4.5) compared with vegetarians (2.9 µg/day; 95% CI: 2.5–3.3; *p* < 0.0001). Supplement use was more common among vegetarians (27%) compared with omnivores (10%). Mean dietary folate intake was higher in vegetarians (227.4 µg/day; 95% CI: 210.1–244.7) than omnivores (206.9 µg/day; 95% CI: 202.4–211.4; *p* = 0.025). Serum vitamin B_12_ concentrations were higher in omnivores (271.3 pmol/L; 95% CI: 260.8–281.7) than vegetarians (231.0 pmol/L; 95% CI: 183.9–278.1; *p* = 0.1). Holotranscobalamin (HoloTC) was significantly lower in vegetarians (39.8 pmol/L; 95% CI: 33.1–46.5) compared with omnivores (63.1 pmol/L; 95% CI: 60.0–66.2; *p* < 0.001). Homocysteine concentrations were significantly higher in vegetarians (11.22 µmol/L; 95% CI: 9.21–13.23) than omnivores (8.49 µmol/L; 95% CI: 8.17–8.81; *p* = 0.009). Serum folate (22.1 vs. 18.1 nmol/L; *p* = 0.170) and red cell folate (583.4 vs. 528.9 nmol/L; *p* = 0.484) did not differ significantly between groups.

**Conclusions:**

Women of childbearing age following vegetarian diets had lower vitamin B_12_ intakes and holotranscobalamin concentrations than omnivores, despite higher supplement use, with a substantial proportion falling below clinically significant thresholds for deficiency and insufficiency. In contrast, folate intake was higher among vegetarians. These findings highlight the need for targeted dietary guidance and supplementation to support adequate vitamin B_12_ status in this population.

**Supplementary Information:**

The online version contains supplementary material available at https://doi.org/10.1007/s00394-026-04117-1.

## Introduction

Vitamin B_12_, also known as cobalamin, is a water-soluble micronutrient that plays a crucial role in various physiological processes, including DNA synthesis, red blood cell formation, and normal neurological function [1]. Maintaining an adequate vitamin B_12_ status is crucial across the life-course, and particularly important for women of childbearing age (WCBA), ages 18–45 years, as it is vital for foetal development and the prevention of neural tube defects during pregnancy [2, 3]. Despite its importance, vitamin B_12_ deficiency remains a significant public health concern across the life-course, with potential consequences such as megaloblastic anaemia, cognitive impairments, and, in severe cases, irreversible neurological damage [4].

Vitamin B_12_ is primarily found in animal-derived foods, such as meat, fish, dairy, and eggs. Consequently, individuals following vegan or vegetarian diets, which exclude or limit animal-derived foods, may require careful dietary planning or use of fortified foods or supplements to ensure adequate vitamin B_12_ intake [5]. Plant-based dietary patterns are adopted for a variety of ethical, environmental, cultural, and health-related reasons. In recent years, interest in vegetarian and plant-forward dietary patterns has grown across many high-income countries, alongside increasing attention to the environmental and health impacts of dietary choices [6, 7]. Plant-based dietary patterns range from vegetarian and vegan diets to more flexible approaches that reduce, but do not eliminate, animal-derived foods. While these dietary patterns can provide many nutritional benefits, ensuring adequate intake of certain micronutrients, including vitamin B_12_, remains an important consideration, particularly for women of childbearing age [8].

Although data is sparse, previous research has indicated that WCBA who adhere predominately to plant-based diets have lower vitamin B_12_ intakes and serum B_12_ levels compared to their omnivore counterparts [9]. This is likely exacerbated by factors such as duration on a vegan/vegetarian diet, inadequate supplementation regimen (dose, formulation), and lack of awareness.

A recent systematic review and meta-analysis by our group evaluated vitamin B_12_ status among adults adhering to vegan diets and found that vegans had significantly lower levels of serum B_12_ and holotranscobalamin (Holo-TC), alongside elevated methylmalonic acid (MMA) and total homocysteine (tHcy) levels compared to omnivores [10]. The results showed no significant differences between vegans and vegetarians for Holo-TC or MMA, but vegans had significantly lower serum B_12_ levels and higher tHcy concentrations compared to vegetarians. Subgroup analyses highlighted the importance of vitamin B_12_ supplementation in ameliorating risk of low B_12_ status, as vegans who used B_12_ supplements exhibited significant improvements in all biomarker concentrations compared to non-supplemented vegans.

### Insights from previous research on folate and vitamin B_12_ status in the UK in WCBA

Recent findings from the National Diet and Nutrition Survey (NDNS) reveal a broader decline in folate status in the UK population between 2008 and 2019. This includes reductions in plasma and RBC folate across age and gender groups, with women of reproductive age particularly impacted 89% of females had RBC folate concentration less than < 748 nmol/L, a threshold that has been identified below which females of reproductive age face an increased risk of developing neural tube defects [11]. According to the study by Jones et al. [12], the proportion of the population meeting recommended folate levels decreased significantly over this period (~ 3% points per year between 2008 and 2019), raising concerns about the adequacy of current dietary practices and public health strategies to address folate deficiency.

In Sukumar et al. [13] provided valuable insights into the vitamin B_12_ status of WCBA in the UK by analysing data from two NDNS datasets (*2000/2001 and 2008–2012)*. The study revealed a notable prevalence of B_12_ deficiency, with 12.4% of women having serum B_12_ levels ≤ 150 pmol/L, despite a median intake of 3.8 µg/day, which exceeded the UK Recommended Nutrient Intake (RNI) of 1.5 µg/day. The authors identified a threshold effect in the relationship between dietary intake and blood levels of B_12_, where intake beyond approximately 5–7 µg/day resulted in stable serum B_12_ concentrations. The study also found high rates of high serum tHcy (≥ 12 µmol/L), highlighting the need for better preconception screening and possibly higher RNIs for B_12_ in the UK. However, gaps remain in understanding how different dietary patterns, particularly vegan and vegetarian diets, affect B_12_ status among WCBA and how adequate supplementation regimens, as well as use of fortified foods, can mitigate B_12_ insufficiency or deficiency in these groups.

### Key research questions to be addressed

This study builds on Sukumar et al. [13] by using updated NDNS data (2008–2019) to examine trends in B_12_ and folate intake and status among UK women of childbearing age. It includes a wider range of B_12_ biomarkers (serum B_12_, HoloTC, tHcy, RBC folate) across diet groups and provides a more detailed analysis of dietary intake, supplementation, and their effects, particularly in light of increasing plant-based diets.

The overarching aim of this study is to assess vitamin B_12_ intake and status among WCBA following vegetarian, and omnivore diets using 2008–2019 data from the NDNS. It aims to examine trends in dietary intake, supplement use, and demographic differences, comparing biomarkers of B_12_ (serum B_12_, HoloTC, homocysteine) and folate (serum folate, RBC folate) across dietary groups.

## Methods

Aim: To examine vitamin B_12_ intake and status, and folate intake and status, between WCBA following omnivore and vegetarian diets in the UK National Diet and Nutrition Survey.

### Objectives

1. To compare vitamin B_12_ intake and biomarker status (serum B_12_, HoloTC, tHcy) and folate intake and biomarker status (RBC folate, serum folate) between omnivores and vegetarians.
2. To assess the impact of supplementation on B_*12*_ intake and biomarker status.
3. To explore predictors of B_*12*_ biomarkers (dietary group, supplementation, BMI, and age).
4. To assess the prevalence of vitamin B_12_ insufficiency and deficiency in WCBA, per diet group.

### Study design and population

This study is a cross-sectional analysis of data from the NDNS covering the years 2008 to 2019. The population of interest includes non-pregnant WCBA between 18 and 45 years with ≥ 4 diary days and available biomarker data, categorised into two dietary groups: omnivore and vegetarian. Diet groups were defined using the self-reported dietary pattern variable collected during the NDNS interview, where participants indicated whether they followed a vegetarian or vegan diet. Participants who did not report following either diet were classified as omnivores. The vegetarian group in this study comprises self-reported lacto-ovo-vegetarians only; for brevity, this group is referred to as vegetarian (Veg) throughout the manuscript (Table 1).

**Table 1:** Demographic characteristics of participants by diet Group in the combined NDNS (2008–2019) in women of childbearing age

Variable	Level	Omnivores (n = 2204)	Vegetarians (n = 89)	p-value
Continuous variables-mean ± SE				
Age (years)	–	32.0 ± 0.2	33.3 ± 1.1	0.227
BMI (kg/m^2^)	–	26.2 (25.9–26.6)	25.8 (24.4–27.3)	0.580
Energy intake (kcal/day)	–	1628.8 (1601.5–1656.0)	1712.9 (1564.3–1861.6)	0.275
Education% (survey-weighted)				
Education	Degree+	33.9%	42.3%	0.173
	Other Higher	10.6%	3.5%	–
	A Levels	16.8%	13.0%	–
	Other	38.7%	41.1%	–
Ethnicity % (survey-weighted)				
Ethnicity	White	83.3%	74.3%	** < 0.001**
	Asian	6.9%	25.0%	–
	Black	4.4%	0.0%	–
	Mixed	2.3%	0.7%	–
	Other	3.0%	0.0%	–
Income tertileᵃ % (survey-weighted)				
Income	Lowest	34.3%	32.1%	0.707
	Middle	30.4%	25.8%	–
	Highest	35.2%	42.1%	–
Smoking status % (survey-weighted)				
Smoking	Current smoker	25.0%	13.5%	**0.018**
	Ex-smoker	15.5%	10.2%	–
	Never smoked	59.5%	76.3%	–

ᵃIncome tertiles: Lowest < £17,500/year; Middle £17,500-£32,000/year; Highest > £32,000/year.

p-values**:** Differences between diet groups were assessed using survey-weighted design-based t-tests for continuous variables (age, BMI, energy intake) and chi-square tests for categorical variables (education, ethnicity, income, smoking status), accounting for the complex survey design. Significant p-values (< 0.05) are shown in bold. Where a p-value is shown only on the first level of a categorical variable, it applies to the overall test for that variable.

As self-reported dietary classification may not always correspond to actual food intake [14, 15], self-reported vegetarian status was cross-referenced against 4-day food diary records for meat and fish intake, and a sensitivity analysis restricting the vegetarian group to those with no reported meat or fish intake in the diary was conducted for the primary comparisons in Tables 2 and 3 (Supplementary Table 1).

**Table 2:** Dietary and total Vitamin B_12_ and Folate intake for diet groups (omnivores and vegetarians) for the combined NDNS years 2008-2019 in WCBA for the total population. Results are shown as mean (95%CI) for omnivores and vegetarians median (IQR) for skewed data

	Omnivores mean (95%CI)	Vegetarians mean (95%CI)	Survey-weighted t-tests (omnivore vs. vegetarian)
N^a^	2204	89	
Dietary B_12_ intake (µg/day)	4.3 (4.2–4.5)	2.9 (2.5–3.3)	** < 0.0001**
Total B_12_ intake (µg/day)	3.9 (2.7–5.4) [median, IQR]	3.2 (2.0–4.6) [median, IQR]	** < 0.003**
N (%) B_12_ supplements	215 (10%)	24 (27%)	** < 0.001**
B_12_ supplement intake only (µg/day)	2.2 (1.0–6.4) [median, IQR]	1.7 (1.0–5.4) [median, IQR]	0.601
Dietary folate intake (µg/day)	206.9 (202.4–211.4)	227.4 (210.1–244.7)	**0.025**
Total folate intake (µg/day)	250.5 (233.9–267.1)	271.4 (241.1–301.7)	** < 0.001**
N (%) Folate supplements	236 (11%)	22 (25%)	** < 0.001**
Folate supplement intake only (µg/day)	386.5 (256.7, 516.3)	213.2 (158.6, 267.8)	0.105

**ᵃ**Total number of participants with dietary data. Number of participants per variable may differ due to missing data.

**ᵇ**Median (IQR) reported due to right-skewed distribution. Wilcoxon rank-sum test used for statistical comparison.

p-values: Survey-weighted t-tests used for normally distributed dietary intake variables. Wilcoxon rank-sum tests used for skewed variables (total B_12_ intake, supplement-only intakes). Chi-square tests used for proportions (supplement use). Significant p-values (< 0.05) are shown in bold.

**Table 3:** Unadjusted and adjusted survey-weighted means of blood biomarkers by diet group in women of childbearing age (18-45 years), NDNS Years 1–11

Outcome	Omn N	Veg N	Omnivore	Vegetarian	Unadjusted difference (95% CI), p-valueᵃ	Omn N	Veg N	Omnivore	Vegetarian	Adjusted difference (95% CI), p-value
Serum B_12_ (pmol/L)	959	41	271.3 (260.8–281.7)	231.0 (183.9–278.1)	− 40.3 (− 88.6–8.0),* p* = 0.102	822	34	289.4 (261.5–317.3)	260.2 (205.7–314.7)	− 29.2 (− 83.5–25.2), *p* = 0.293
Holotranscobalamin (pmol/L)	452	21	63.1 (60.0–66.2)	39.8 (33.1–46.5)	**− 23.3 (− 30.7**–** −15.9), *p* = < 0.001**	381	17	61.9 (56.7–67.1)	40.4 (31.1–49.6)	**− 21.5 (-30.6**–** −12.5), *p* = < 0.001**
Red cell folate (nmol/L)	926	41	528.9 (507.6–550.3)	583.4 (432.4–734.5)	+ 54.5 (− 98.2–207.2),* p* = 0.484	794	33	546.5 (511.6–581.4)	552.4 (456.6–648.3)	+6.0 (-86.2–98.1),* p* = 0.899
Serum folate (nmol/L)	967	42	18.1 (16.0–20.2)	22.1 (16.8–27.3)	+ 4.0 (− 1.7–9.6),* p* = 0.170	831	34	17.6 (15.9–19.3)	18.8 (14.3–23.3)	+1.2 (− 3.5–5.9),* p* = 0.620
Homocysteine (µmol/L)ᵃ	507	18	8.49 (8.17–8.81)	11.22 (9.21–13.23)	**+ 2.73 (0.69**–**4.77), *p* = 0.009**	440	15	7.95 (7.54–8.39)	11.80 (9.73–14.32)	**Ratio: 1.485 (1.234**–**1.786), *p* = < 0.001**

Significant p-values (<0.05) shown in bold.

**ᵃ**Homocysteine was log-transformed prior to modelling due to right-skewed distribution; the unadjusted difference is shown on the original scale for comparability. Back-transformed geometric mean ratios are reported for the adjusted difference. A ratio >1 indicates higher homocysteine in vegetarians.

Confounder-adjusted marginal means estimated using weighted linear regression, adjusting for age, ethnicity, education, income, and BMI. Supplement use was excluded from B_12_ biomarker models to avoid overadjustment. Adjusted models were restricted to participants with complete data on all covariates (age, ethnicity, education, income, and BMI); adjusted sample sizes are therefore smaller than the unadjusted sample sizes shown, and unadjusted and adjusted estimates should not be interpreted as derived from the same underlying sample.

The survey programme’s background, funding, structure, and accessible data have been detailed in publications [16], with survey findings and reports also publicly available [17]. In brief, dietary and questionnaire data were collected annually from approximately 500 adults (aged ≥ 19 years) and 500 children (aged 1.5 to 18 years) residing across the United Kingdom (England, Wales, Scotland, and Northern Ireland). Each year, around 500 participants also provided blood samples for nutritional biomarker analysis. An overview of the survey’s sampling methodology can be found in [16], with further specifics provided in the reports and appendices for each survey cycle [17]. The NDNS employs a rolling cross-sectional design with independent samples each year, repeated measures in this analysis represent pooled survey years rather than longitudinal follow-up of the same individuals.

The NDNS Rolling Programme adheres to the principles of the Declaration of Helsinki and has received ethical approval from the UK NHS Health Research Authority Research Ethics Committees (Years 1–5: Oxfordshire REC A, REF 07/H0604/113; Years 6–10 and 11–15: East of England - Cambridgeshire South REC, REF 13/EE/0016).

This report presents data from the NDNS covering the years 2008 to 2019, grouped by survey periods: Years 1–4 (2008/09-2011/12), Years 5–6 (2012/13-2013/14), Years 7–8 (2014/15-2015/16), and Years 9–11 (2016/17-2018/19).

### Dietary sample collection

Dietary intake of vitamin B_12_ and folate was assessed using 4-day estimated food diaries collected as part of the NDNS. Participants recorded all foods, beverages, and dietary supplements consumed over four consecutive days. Intake from both dietary sources and supplements was included.

Nutrient intakes were calculated by NDNS using food composition data from the UK Nutrient Databank. Vitamin B_12_ intake (µg/day) and folate intake (µg/day) were derived from the databank. Dietary supplement use was recorded within the food diaries and included both supplement use (yes/no) and nutrient intake from supplements where available. Food diaries were manually coded using a customised dietary analysis system and linked to the NDNS nutrient databank, which incorporates data from the Composition of Foods Integrated Dataset, the FSA Food Recipes Database, and product information obtained from food packaging and online sources [16]. Data collection was distributed across all days of the week to ensure balanced representation.

### Dietary thresholds

The following recommended nutrient intakes (RNI) were utilised to evaluate adequacy of intake of the micronutrients under study: For vitamin B_12_ intake the UK RNI 1.5 mcg/day [18] and the EFSA adequate intake (AI) of 4 mcg/day [19]. For folate intake the UK RNI is 200 mcg/day for adults [18] and the EFSA AI of 250 mcg/day [20].

### Biomarker sample collection

Vitamin B_12_ was measured in serum using the Siemens Advia Centaur analyser, which applies the manufacturer’s competitive protein binding method, with analyses conducted at Addenbrooke’s Hospital. HoloTC was also measured in serum using an ELISA assay (Axis Shield Diagnostics, Dundee) during Years 6–11 of the survey. Plasma tHcy concentrations were assessed using an immunoturbidimetric assay on the Siemens BN ProSpec analyser (manufacturer’s method) in Years 1–5. Serum folate was measured using liquid chromatography-tandem mass spectrometry (LC-MS/MS). Whole blood folate was determined using a microbiological assay, also performed at the CDC; red cell folate values were subsequently calculated using whole blood folate, serum folate, and haematocrit measurements. Further information about the blood sample collection, processing, storage and analysis are describe in prior work [16].

### Biomarker thresholds

Biomarkers analysed include serum B_12_, HoloTC, tHcy, serum folate, and RBC folate. HoloTC and tHcy were measured in three NDNS datasets, with HoloTC absent in the 2008–2010 dataset and tHcy in the 2016–2019 dataset. The following biomarkers and thresholds, based on the National Institute for Health and Care Excellence (NICE) guidelines and other established standards, were used:

- Serum B_12_ Severe deficiency: < 133 pmol/L [21], Deficiency: < 180 pmol/L, Potential deficiency: < 258 pmol/L [21].
- HoloTC Severe deficiency: < 25 pmol/L [21], Deficiency: < 36 pmol/L [22, 23], and Potential deficiency: < 70 pmol/L [21].
- Total Homocysteine elevated levels indicating deficiency: >15 µmol/L.
- A RBC folate concentration < 748 nmol/L threshold below which females of reproductive age are at increased risk of neural tube defects A lower threshold of < 305 nmol/L indicates folate deficiency and elevated risk of megaloblastic anaemia [11].
- Serum folate: < 7 nmol/L and < 13 nmol/L represent deficiency and possible deficiency in relation to risk of megaloblastic anaemia, respectively [24].

### Data quality control

NDNS applies rigorous data cleaning and validation procedures before release. For the present analyses, participants with missing biomarker or weight data were excluded. Implausible values (extreme intakes > 99th percentile or biologically implausible biomarker concentrations flagged by NDNS quality checks) were excluded. For supplement intake, zero values were retained as “non-users”; missing values were set to missing rather than zero to avoid misclassification.

### Survey weights

Survey weights were applied to all analyses following NDNS guidance to ensure national representativeness. Pooled weights for combined survey Years 1–11 were created using NDNS methods to account for differences in sampling probabilities and non-response across survey years.

For subgroup analyses restricted to WCBA with available biomarker data, weights were re-scaled to the analytic sample in accordance with NDNS documentation. Separate weights were used for dietary intake variables and blood biomarker analyses. Weighted analyses were conducted in R (version 4.3.0) using the survey package.

### Statistical analysis

Dietary and biomarker data were summarised by dietary group (omnivore, vegetarian) and by vitamin B_12_ supplement status. Normally distributed variables were presented as survey-weighted means with 95% confidence intervals (95% CI). Skewed variables (total B_12_ intake, supplement-only intakes) were presented as medians with interquartile ranges (IQR). Between-group differences for continuous variables were assessed using survey-weighted design-based t-tests. For categorical variables and prevalence comparisons, Chi-square tests were used, accounting for the complex survey design. Wilcoxon rank-sum tests were used where distributions were non-normal.

For temporal trend analysis, NDNS survey year was modelled as a continuous variable (Years 1–11) and the annual change (*β* per year, 95% CI, p-value) for each dietary intake and biomarker was estimated within each diet group using survey-weighted linear regression.

To compare vitamin B_12_ and folate biomarker status between omnivores and vegetarians, both unadjusted survey-weighted means and confounder-adjusted marginal means were estimated. Adjusted marginal means were derived from weighted linear regression models. Models were adjusted for age, ethnicity, education, income, and BMI. Supplement use was deliberately excluded from B_12_ biomarker models to avoid overadjustment, as it lies on the causal pathway between dietary pattern and B_12_ status. Homocysteine was log-transformed prior to modelling due to right-skewed distribution; back-transformed geometric mean ratios are reported.

Multivariable linear regression models were fitted within the survey design to examine predictors of vitamin B_12_ and folate biomarkers. Predictors included dietary group, B_12_ supplement use, B_12_ and folate dietary intakes, energy intake (kcal/day), age, ethnicity, education, and household income. Smoking status was initially considered but excluded due to high levels of missing data. Models were also run separately for omnivores and vegetarians as sensitivity analyses; these results are presented in the Supplementary Material. Model fit was assessed using R^2^.

Weighted prevalence estimates of low vitamin B_12,_ and folate status were calculated for women aged 18–45 years, stratified by dietary group. Deficiency and suboptimal status were defined using established biochemical cut-offs for serum B_12_, holoTC, RBC folate, serum folate, and total homocysteine. Dietary adequacy was assessed against UK EAR, RNI, and EFSA AI values. Prevalence estimates and 95% CIs were calculated using survey-weighted analyses in R, applying NDNS dietary and blood weights to account for sampling design and non-response. All statistical analyses were conducted in R (version 4.5). Statistical significance was set at* α* = 0.05.

### Justification to pool the data

To support robust estimation and enable subgroup analyses, data from multiple survey cycles 2008–2019 were combined. This pooling enhances statistical power, particularly for analyses involving smaller subgroups, and is explicitly recommended in NDNS programme materials and previous research. The continuous nature of the NDNS facilitates such analyses, making it possible to examine both overall trends and more granular differences by population subgroups. "*The collection of detailed anthropometric*,* sociodemographic*,* economic and other contextual and behavioural information enables analysis by population sub-groups when data years are combined (pg 9). "* [16].

## Results

Initial analysis examining trends across successive NDNS survey years from 2008 to 2019 (Supplementary Table 2) showed that among omnivores, vitamin B_12_ intake did not differ significantly across surveys (*β* = 0.032, 95% CI: − 0.015 to 0.078, *p* = 0.18). The surveys reported a significant declining trend in folate intake over the years (*β* = − 2.144, 95% CI: − 3.270 to − 1.017, *p* < 0.001); B_12_ supplement intake showed an increasing trend across surveys (*β* = 2.746, 95% CI: 0.912 to 4.581, *p* = 0.003), while folate supplement intake showed no significant trend across surveys (*β* = 7.294, 95% CI: − 29.875 to 44.463, *p* = 0.70). For biomarkers, serum vitamin B_12_ showed an increasing trend across surveys (*β* = 5.504, 95% CI: 3.070 to 7.938, *p* < 0.001), HoloTC did not differ significantly across surveys (*β* = − 0.040, 95% CI: − 1.521 to 1.440, *p* = 0.96), RBC folate showed a declining trend across surveys (*β* = − 13.107, 95% CI: − 18.875 to − 7.338, *p* < 0.001), and serum folate did not differ significantly across surveys (*β* = − 0.018, 95% CI: -0.648 to 0.612, *p* = 0.96).

Among vegetarians, no significant trends across surveys were observed in vitamin B_12_ intake (*β* = 0.067, 95% CI: − 0.051 to 0.186, *p* = 0.27), folate intake (*β* = − 2.492, 95% CI: − 8.380 to 3.396, *p* = 0.41), B_12_ supplement intake (*β* = 5.752, 95% CI: − 6.120 to 17.624, *p* = 0.35), or folate supplement intake (*β* = 6.583, 95% CI: − 14.138 to 27.304, *p* = 0.54). Serum vitamin B_12_ showed a borderline declining trend across surveys (*β* = − 17.092, 95% CI: − 34.258 to 0.074, *p* = 0.06), while HoloTC (*β* = − 2.505, 95% CI: − 6.039 to 1.030, *p* = 0.18), RBC folate (*β* = 24.651, 95% CI: − 20.193 to 69.495, *p* = 0.29), and serum folate (*β* = 0.941, 95% CI: − 0.790 to 2.673, *p* = 0.29) did not show significant trends across surveys.

Figures of heatmaps for B_12_ intake, Folate intake, Serum B_12_, HoloTC, RBC folate and Serum Folate can be found in the Supplementary Material Fig. 1A–F.

### Summary of the combined NDNS data for WCBA (Years 2008–2019) for dietary intake and biomarkers

Overall, the NDNS data from 2008 to 2019 included 2,299 WCBA of whom 2,204 were omnivores and 89 were vegetarians. Cross-referencing self-reported vegetarian status against 4-day food diary records identified that 22 of the 89 self-reported vegetarians (24.7%) recorded meat and/or fish intake in their diary (15 fish-only, 7 meat-only, none reporting both). A sensitivity analysis restricting the vegetarian group to the 67 participants with no reported meat or fish intake yielded consistent findings across all dietary intake and biomarker comparisons reported in Tables 2 and 3, with no outcome changing in direction or statistical significance (Supplementary Table 1). Summary characteristics of the pooled dietary groups are presented in Table 1. The majority of participants were White (83–100%), with smaller proportions from Mixed, Black, Asian, and Other ethnic backgrounds. Asian representation was higher among vegetarians (25%) compared with omnivores (7%). Smoking prevalence was greatest among omnivores (25% current smokers) compared with vegetarians (14%). Educational attainment showed broadly similar distributions overall, a degree-level qualification was reported by 42% of vegetarians and 34% of omnivores.

### Dietary and supplemental vitamin B_12_ and folate intakes in WCBA across NDNS years 2008–2019

For dietary vitamin B_12_ intake, omnivores had a weighted mean of 4.3 µg/day (95% CI: 4.2–4.5), vegetarians 2.9 µg/day (95% CI: 2.5–3.3). The survey-weighted t-test indicated a significant difference between omnivores and vegetarians (*p* < 0.0001) Table 2.

For total vitamin B_12_ intake (diet plus supplements), the median (IQR) was 3.9 µg/day (2.7–5.4) in omnivores and 3.2 µg/day (2.0–4.6) in vegetarians. The proportion of participants reporting B_12_ supplement use was 215 (10%) among omnivores and 24 (27%) among vegetarians. Among supplement users, the median (IQR) intake from supplements only was 2.2 µg/day (1.0–6.4) for omnivores and 1.7 µg/day (1.0–5.4) for vegetarians.

For dietary folate intake, omnivores had a weighted mean of 206.9 µg/day (95% CI: 202.4–211.4) and vegetarians 227.4 µg/day (95% CI: 210.1–244.7). The survey-weighted t-test showed a significant difference between omnivores and vegetarians (*p* = 0.025).

For total folate intake, omnivores had a weighted mean of 250.5 µg/day (95% CI: 233.9–267.1) and vegetarians 271.4 µg/day (95% CI: 241.1–301.7). Reported supplement use was 236 omnivores and 22 vegetarians. Among supplement users, folate intake from supplements only was 386.5 µg/day (95% CI: 256.7–516.3) for omnivores and 213.2 µg/day (95% CI: 158.6–267.8) for vegetarians.

### Biomarker concentrations by dietary group in women of childbearing age

Unadjusted and confounder-adjusted means are presented in Table 3. Among omnivores, the unadjusted mean (95% CI) serum B_12_ concentration was 271.3 pmol/L (260.8–281.7), compared with 231.0 pmol/L (183.9–278.1) in vegetarians (difference: − 40.3 pmol/L, 95% CI − 88.6−8.0, *p* = 0.102). Following adjustment for age, ethnicity, education, income, and BMI, the difference was attenuated but remained similar in magnitude (− 29.2 pmol/L, 95% CI − 83.5–25.2, *p* = 0.293).

For HoloTC, omnivores had an unadjusted mean of 63.1 pmol/L (60.0–66.2) compared with 39.8 pmol/L (33.1–46.5) in vegetarians (difference: − 23.3 pmol/L, 95% CI − 30.7– −15.9, *p* < 0.001). This difference was robust to confounder adjustment (adjusted difference: − 21.5 pmol/L, 95% CI − 30.6– −12.5, *p* < 0.001), indicating that demographic differences between groups explain little of the observed difference in HoloTC.

Mean RBC folate was 528.9 nmol/L (507.6–550.3) in omnivores and 583.4 nmol/L (432.4–734.5) in vegetarians (difference: + 54.5 nmol/L, 95% CI − 98.2–207.2, *p* = 0.484). Following adjustment, this difference attenuated substantially (+ 6.0 nmol/L, 95% CI − 86.2−98.1, *p* = 0.899), suggesting the unadjusted difference was largely explained by demographic confounding. Serum folate concentrations were 18.1 nmol/L (16.0–0.2) in omnivores and 22.1 nmol/L (16.8–27.3) in vegetarians (difference: + 4.0 nmol/L, 95% CI − 1.7–9.6, *p* = 0.170), with no significant difference after adjustment (+ 1.2 nmol/L, 95% CI − 3.5–5.9, *p* = 0.620).

Homocysteine was significantly higher in vegetarians compared with omnivores (11.22 µmol/L, 95% CI 9.21–13.23 vs. 8.49 µmol/L, 95% CI 8.17–8.81; difference: + 2.73 µmol/L, 95% CI 0.69–4.77, *p* = 0.009). Following log-transformation and confounder adjustment, vegetarians had a geometric mean homocysteine 1.49 times higher than omnivores (95% CI 1.23–1.79, *p* < 0.001), confirming this finding was robust to adjustment.

### Stratified dietary and total vitamin B_12_ intake and biomarker levels for WCBA categorised by vitamin B_12_ supplement use

Among omnivores, B_12_ supplement use was associated with higher dietary B_12_ intake (4.7 µg/day vs. 4.3 µg/day, *p* = 0.16) and substantially higher total intake (median 7.4 [5.0–11.0] µg/day vs. 3.7 [2.6-5.0] µg/day, *p* < 0.001, Supplementary Table 3). Supplement users also had higher folate intake (227.0 vs. 204.7 µg/day, *p* = 0.002), and significantly higher circulating biomarkers including serum B_12_ (332.4 vs. 262.9 pmol/L, *p* = 0.015), holoTC (75.2 vs. 61.1 pmol/L, *p* = 0.001), red cell folate (724.6 vs. 503.6 nmol/L, *p* < 0.001), and serum folate (28.1 vs. 16.7 nmol/L, *p* < 0.001).

In vegetarians, sample sizes for supplement users were much smaller (*n* = 24), and biomarker subsamples were limited (*n* ≤ 12). As such, estimates should be interpreted cautiously. Nevertheless, supplement use among vegetarians appeared to increase total B_12_ intake, though mean serum B_12_ and holoTC concentrations remained lower than in omnivores. Folate biomarkers (serum and red cell folate) were generally higher in supplementing vegetarians compared to non-supplementers, though confidence intervals were wide. Results stratified by supplement use for dietary intake and biomarker concentrations among vegetarians are presented in Supplementary Table 3.

### Identifying predictors of vitamin B_12_ biomarkers among omnivore and vegetarian WCBA

Multivariable regression models were fitted to examine predictors of vitamin B_12_ and folate biomarkers among women of childbearing age, including omnivores and vegetarians (Table 4).

**Table 4:** Multiple regression models for predictors of blood biomarkers (weighted, females 18-45y, NDNS 2008-2019). Results are presented as *β *(95% CI). Significant p-values (< 0.05) are shown in bold red

Predictor	Serum B_12_	HoloTC	RBC Folate	Serum folate
N	856	398	820	856
R^2^	0.098	0.081	0.071	0.089
Intercept	228.12 (155.84, 300.39)	63.65 (41.13, 86.17)	337.51 (182.97, 492.05)	12.07 (4.20, 19.95)
p-value	** < 0.001**	** < 0.001**	** < 0.001**	**0.003**
Diet: Vegetarian (vs Omnivore)	− 34.05 (− 91.30, 23.19)	− 22.40 (− 31.89, − 12.91)	38.52 (− 51.42, 128.46)	2.89 (− 2.30, 8.07)
p-value	0.243	** < 0.001**	0.400	0.274
B_12_ supplement use (Yes vs No)	63.33 (12.97, 113.69)	9.77 (2.19, 17.35)	228.58 (136.13, 321.02)	11.42 (6.24, 16.60)
p-value	**0.014**	**0.012**	** < 0.001**	** < 0.001**
Age (per year)	1.94 (0.53, 3.36)	0.07 (− 0.33, 0.47)	1.19 (− 1.71, 4.09)	− 0.10 (− 0.29, 0.09)
p-value	**0.007**	0.724	0.421	0.302
Ethnicity: Mixed (vs White)	51.71 (1.62, 101.79)	− 1.93 (− 11.51, 7.66)	− 76.54 (− 194.65, 41.57)	− 0.79 (− 6.85, 5.28)
p-value	**0.043**	0.693	0.205	0.800
Ethnicity: Black (vs White)	291.47 (45.18, 537.76)	27.89 (− 2.35, 58.13)	86.25 (− 120.86, 293.36)	0.22 (− 5.19, 5.63)
p-value	**0.021**	0.071	0.414	0.937
Ethnicity: Asian (vs White)	7.94 (− 33.92, 49.80)	4.52 (− 11.48, 20.52)	42.97 (− 50.56, 136.49)	− 1.74 (− 6.30, 2.83)
p-value	0.710	0.580	0.369	0.455
Ethnicity: Other (vs White)	76.86 (37.93, 115.78)	8.24 (− 7.61, 24.10)	72.41 (− 44.34, 189.16)	3.36 (− 7.13, 13.84)
p-value	** < 0.001**	0.307	0.224	0.530
Education: Other Higher (vs Degree+)	− 23.11 (− 45.44, − 0.77)	− 5.11 (− 13.03, 2.81)	14.88 (− 52.76, 82.52)	− 0.07 (− 3.48, 3.34)
p-value	**0.043**	0.205	0.666	0.968
Education: A Levels (vs Degree+)	41.66 (− 5.23, 88.56)	− 2.37 (− 12.50, 7.76)	− 23.17 (− 86.25, 39.91)	− 2.63 (− 6.34, 1.08)
p-value	0.082	0.645	0.472	0.165
Education: Other (vs Degree+)	5.87 (− 17.55, 29.29)	0.63 (− 7.29, 8.55)	− 13.86 (− 79.35, 51.63)	2.06 (− 9.01, 13.12)
p-value	0.623	0.876	0.678	0.716
Income: Middle (vs Lowest)	− 36.78 (− 65.94, − 7.61)	− 10.74 (− 18.97, − 2.51)	20.23 (− 40.34, 80.79)	− 2.14 (− 11.05, 6.78)
p-value	**0.014**	**0.011**	0.512	0.638
Income: Highest (vs Lowest)	− 5.36 (− 37.72, 27.00)	− 0.43 (− 9.80, 8.93)	24.15 (− 34.16, 82.47)	0.03 (− 7.76, 7.82)
p-value	0.745	0.928	0.418	0.994
BMI (per unit)	− 1.63 (− 3.45, 0.19)	− 0.01 (− 0.57, 0.55)	− 2.95 (− 7.25, 1.35)	− 0.06 (− 0.33, 0.20)
p-value	0.079	0.972	0.178	0.636
Energy intake (kcal/day)	0.01 (− 0.01, 0.03)	0.00 (− 0.01, 0.01)	0.04 (− 0.01, 0.09)	0.00 (− 0.00, 0.01)
p-value	0.463	0.967	0.118	0.487

For serum vitamin B_12_, significant predictors included B_12_ supplement use (*β* = 68.1 pmol/L, 95% CI: 19.1, 117.0, *p* = 0.007), age (*β* = 1.89 pmol/L per year, 95% CI: 0.50, 3.29, *p* = 0.008), and dietary B_12_ intake (*β* = 10.8 pmol/L per µg/day, 95% CI: 6.39, 15.3, *p* < 0.001). Ethnicity was also associated, with higher of B_12_ concentrations observed in participants identifying as Black (*β* = 289.1 pmol/L, 95% CI: 37.2, 541.1, *p* = 0.025) and other ethnic backgrounds (*β*  = 77.4 pmol/L, 95% CI: 38.0, 116.8, *p* < 0.001) compared to White participants.

For holoTC, both B_12_ supplement use (*β* = 12.3 pmol/L, 95% CI: 3.75, 20.9, *p* = 0.005) and dietary B_12_ intake (*β* = 3.86 pmol/L per µg/day, 95% CI: 1.97, 5.75, *p* < 0.001) were significant predictors.

For red cell folate, folate intake (*β* = 0.80 nmol/L per µg/day, 95% CI: 0.44, 1.17, *p* < 0.001) and B_12_ supplement use (*β* = 226.1 nmol/L, 95% CI: 135.3, 316.8, *p* < 0.001) were strongly associated with higher concentrations.

For serum folate, both folate intake (*β* = 0.05 nmol/L per µg/day, 95% CI: 0.03, 0.07, *p* < 0.001) and B_12_ supplement use (*β* = 11.6 nmol/L, 95% CI: 6.49, 16.8, *p* < 0.001) were significant predictors.

Regression models including omnivores and vegetarians combined are presented here. Stratified models by dietary group are provided in the Supplementary Tables 4 and 5, given the small sample size among vegetarians and the wide confidence intervals observed.

Model fit was modest across all four biomarkers, with R^2^ ranging from 0.071 to 0.098 (Table 4), indicating that the measured predictors accounted for a small proportion of the total variance in biomarker concentrations. Individual predictors identified above should therefore be interpreted as statistically significant associations within these models, rather than as evidence of strong overall predictive capacity.

### Prevalence of dietary vitamin B_12_ deficiency according to the EFSA and UK guidelines in women of childbearing age

The data presented in Table 5 highlights the proportion of WCBA adhering to omnivore and vegetarian who meet the recommended vitamin B_12_ intake levels according to the UK RNI of 1.5 µg/day and the EFSA AI of 4 µg/day. The results are stratified by dietary group and further categorised into dietary B_12_ intake alone and total B_12_ intake, which includes supplementation.

**Table 5:** Proportion (%) of omnivore and vegetarian women of childbearing age (WCBA, 18–45 years) below vitamin B_12_ and folate intake cut-offs according to UK and EFSA dietary recommendations, combined NDNS 2008-2019. Results are shown as survey-weighted % (95% CI)

Dietary recommendation cut-off	Omnivores % (95% CI)	Vegetarians % (95% CI)	p-valueᵃ
Vitamin B_12_ -dietary intake			
Dietary B_12_ intake < UK EAR (1.25 µg/day)	3.3 (2.4–4.3)	12.1 (3.3–21.0)	** < 0.001**
Dietary B_12_ intake < UK RNI (1.5 µg/day)	5.3 (4.0–6.5)	21.8 (9.7–33.8)	** < 0.001**
Dietary B_12_ intake < EFSA AI (4 µg/day)	51.7 (49.0–54.5)	78.2 (67.4–89.1)	** < 0.001**
Vitamin B_12_ -total intake (diet + supplements)ᵇ			
Total B_12_ intake < UK EAR (1.25 µg/day)	2.8 (1.9–3.7)	5.8 (− 0.1–11.6)	0.182
Total B_12_ intake < UK RNI (1.5 µg/day)	4.7 (3.5–5.8)	14.0 (3.3–24.6)	**0.008**
Total B_12_ intake < EFSA AI (4 µg/day)	49.2 (46.4–51.9)	65.9 (53.7–78.0)	**0.013**
Folate-dietary intake			
Dietary folate intake < UK RNI (200 µg/day)	51.7 (48.9–54.4)	39.0 (25.7–52.2)	0.072
Dietary folate intake < EFSA AI (250 µg/day)	74.5 (72.0–76.9)	65.8 (53.8–77.8)	0.137
Folate - total intake (diet + supplements)ᵇ			
Total folate intake < UK RNI (200 µg/day)	47.0 (44.3–49.8)	35.5 (22.2–48.8)	0.110
Total folate intake < EFSA AI (250 µg/day)	68.0 (65.4–70.6)	53.0 (40.1–65.9)	**0.018**

**ᵃ**P-values from chi-square tests comparing proportions below each cut-off between omnivores and vegetarians. Significant p-values (<0.05) are shown in bold red.

**ᵇ**Total intake includes dietary sources and supplements combined.

EAR: Estimated Average Requirement. RNI: Reference Nutrient Intake. EFSA AI: European Food Safety Authority Adequate Intake.

Negative lower CI limits for small subgroups (e.g., vegetarians below EAR) reflect small sample sizes and should be interpreted with caution.

Among WCBA, 3.3% (95% CI: 2.4–4.3) of omnivores and 12.1% (3.3–21.0) of vegetarians had dietary vitamin B_12_ intakes below the UK Estimated Average Requirement (EAR; 1.25 µg/day). A higher proportion fell below the UK RNI (1.5 µg/day), at 5.3% (4.0–6.5) in omnivores and 21.8% (9.7–33.8) in vegetarians. More than half of omnivores (51.7% [49.0–54.5]) and over three-quarters of vegetarians (78.2% [67.4–89.1]) had dietary vitamin B_12_ intakes below the EFSA Adequate Intake (AI; 4 µg/day).

When considering total vitamin B_12_ intake (food plus supplements), the prevalence of inadequacy was slightly lower across all cut-offs. Among WCBA omnivores, 2.8% (1.9–3.7) and 4.7% (3.5–5.8) were below the UK EAR and RNI, respectively, compared with 5.8% (− 0.1–11.6) and 14.0% (3.3–24.6) of vegetarians. Half of omnivores (49.2% [46.4–51.9]) and two-thirds of vegetarians (65.9% [53.7–78.0]) remained below the EFSA AI threshold despite supplement use.

For folate intake, approximately half of WCBA omnivores (51.7% [48.9–54.4]) and 39.0% (25.7–52.2) of vegetarians had dietary intakes below the UK RNI (200 µg/day), while three-quarters of omnivores (74.5% [72.0–76.9]) and two-thirds of vegetarians (65.8% [53.8–77.8]) were below the EFSA AI (250 µg/day).

### Prevalence of vitamin B_12_ deficiency and insufficiency biomarkers in women of childbearing age

**Fig. 1:**
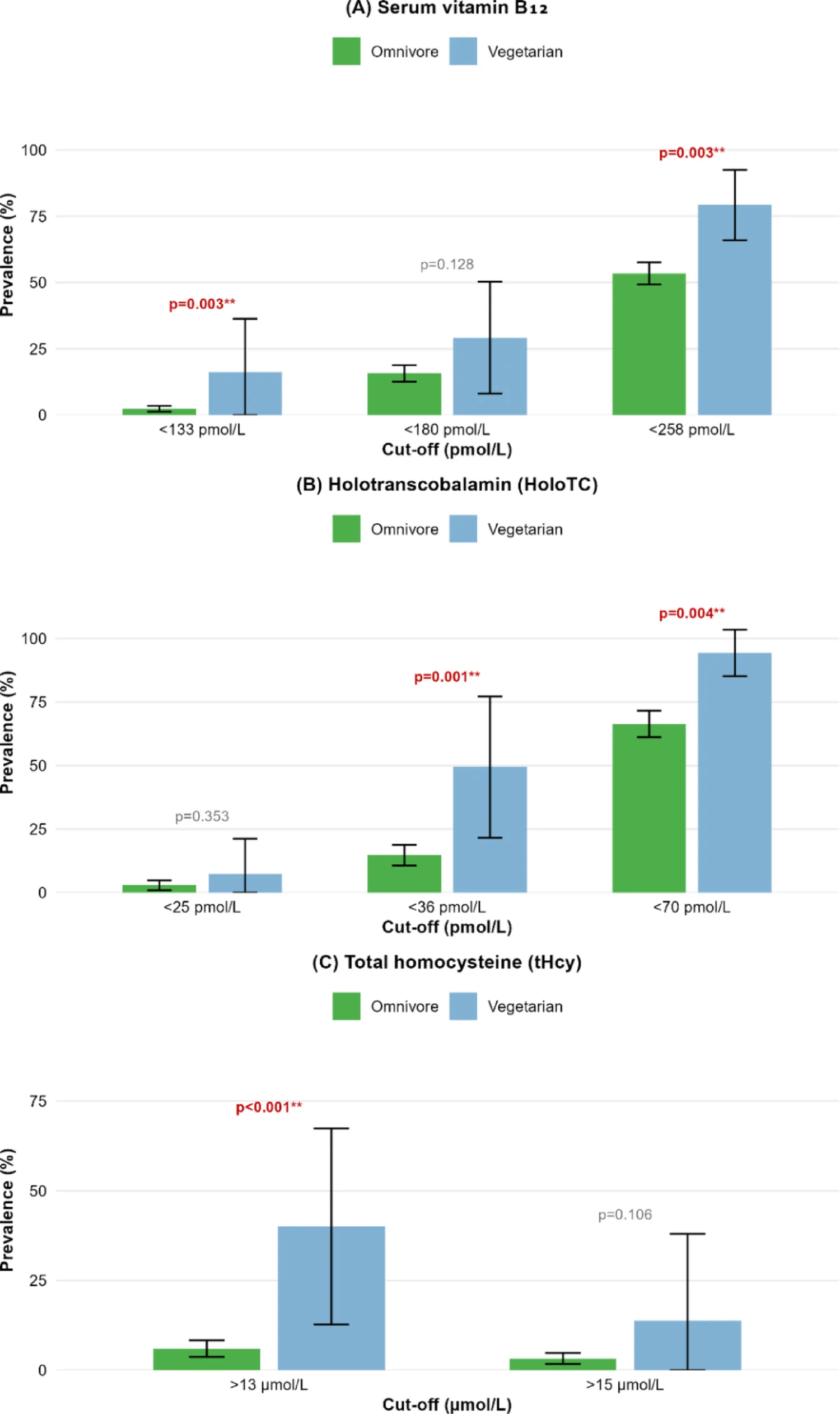
Prevalence (%) of suboptimal vitamin B_12_ status among omnivore and vegetarian women of childbearing age (18–45 years), combined NDNS 2008–2019. **A** Serum vitamin B_12_ using three cut-offs: severe deficiency (< 133 pmol/L), deficiency (< 180 pmol/L), and possible deficiency/insufficiency (< 258 pmol/L). **B** Holotranscobalamin (HoloTC) using three cut-offs: severe deficiency (< 25 pmol/L), low HoloTC (< 36 pmol/L), and possible deficiency/insufficiency (< 70 pmol/L). **C** Total homocysteine (tHcy): elevated homocysteine (> 13 µmol/L) and hyperhomocysteinaemia (> 15 µmol/L). Error bars represent 95% confidence intervals. Negative lower confidence limits for vegetarian subgroups reflect small sample sizes and are shown as zero. Significant differences (*p* < 0.05) are shown in bold red

Biomarker data for serum Vitamin B_12_, holoTC, RBC folate, serum folate, and tHcy are presented in Supplementary Table 6. Among women of childbearing age (WCBA; 18–45 years), 2.4% (95% CI: 1.3–3.5) of omnivores and 16.2% (− 3.9–36.3) of vegetarians had serum vitamin B₁₂ concentrations below 133 pmol/L, indicative of severe deficiency Fig. 1a. The prevalence of vitamin B_12_ deficiency (< 180 pmol/L) was 15.7% (12.6–18.8) in omnivores and 29.2% (8.1–50.3) in vegetarians. More than half of omnivores (53.4% [49.3–57.6]) and nearly four-fifths of vegetarians (79.2% [65.9–92.5]) had serum vitamin B_12_ concentrations below 258 pmol/L, the threshold for possible deficiency.

Patterns were similar for holoTC, a sensitive marker of Vitamin B_12_ status. Among WCBA, 2.9% (0.9–4.8) of omnivores and 7.3% (− 6.6–21.2) of vegetarians had holoTC concentrations below 25 pmol/L, indicative of severe deficiency. The prevalence of possible deficiency (< 70 pmol/L) was markedly higher-66.4% (61.2–71.6) in omnivores and 94.3% (85.2–103.5) in vegetarians Fig. 1b.

For folate biomarkers, 13.4% (10.5–16.4) of WCBA omnivores and 5.0% (− 2.0–12.0) of vegetarians had red cell folate concentrations below 305 nmol/L, indicative of folate deficiency. The majority of women were below the 748 nmol/L threshold associated with increased neural tube defect (NTD) risk-85.0% (81.9–88.1) in omnivores and 89.6% (80.5–98.7) in vegetarians.

Serum folate results followed a similar pattern. Among WCBA, 9.4% (6.9–11.9) of omnivores and 4.8% (− 4.3–13.9) of vegetarians were below 7 nmol/L, while 46.4% (42.2–50.5) and 29.4% (11.7–47.0) were below 13 nmol/L, respectively.

Conversely, total homocysteine (tHcy) concentrations above the deficiency thresholds were uncommon. Among WCBA, 93.5% (91.1–95.9) of omnivores and 60.0% (32.6–87.3) of vegetarians had tHcy concentrations ≤ 13 µmol/L, while 96.7% (95.2–98.3) and 86.2% (62.0–110.4), respectively, were ≤ 15 µmol/L Fig. 1c.

## Discussion

Vitamin B_12_ and folate are crucial micronutrients for WCBA, with deficiencies potentially leading to severe health consequences, including neural tube defects in newborns [3]. The growing adoption of plant-based diets has increased interest in understanding the nutritional profiles associated with these dietary patterns, particularly with respect to vitamin B_12_ intake [25].

This study examined trends in vitamin B_12_ and folate intakes from both diet and supplements among WCBA following vegetarian, and omnivore diets in the UK, using NDNS data from 2008 to 2019. It assessed how these dietary patterns influenced static and functional (serum B_12_, HoloTC, tHcy, serum folate and RBC folate) biomarkers of B_12_ and folate status across dietary groups. Lastly, the study investigated key predictors of B_12_ biomarker levels among omnivores and vegetarians.

### Vitamin B_12_ and folate Intake and biomarker status across dietary groups

Vitamin B_12_ intake from food was highest among omnivores and lowest among vegetarians, consistent with previous NDNS-based and international studies [13, 26]. Despite higher B_12_ supplement use among vegetarians (27% vs. 10%, *p* < 0.001), 65.9% remained below the EFSA adequate intake of 4 µg/day even after including supplements, suggesting that supplementation in this group is inconsistent or insufficiently dosed.

These dietary differences were reflected in biomarker status. Holotranscobalamin was significantly lower in vegetarians both before and after confounder adjustment (*p* < 0.001 for both), and the negligible attenuation following adjustment suggests this reflects dietary pattern rather than demographic differences between groups. Serum B_12_ did not differ significantly between omnivores and vegetarians either before or after adjustment (unadjusted *p* = 0.102; adjusted *p* = 0.293) though the point estimate for this difference should be interpreted with caution, as the adjusted and unadjusted estimates are not derived from identical samples. The persistence of significantly elevated tHcy after adjustment provides the most compelling evidence of functional B_12_ inadequacy, representing a downstream metabolic consequence rather than simply a low blood level. Together these findings suggest that vegetarian WCBA face a metabolic risk of B_12_ inadequacy, and that HoloTC and tHcy together provide a more complete picture of B_12_ status than serum B_12_ alone.

The observed attenuation for serum B_12_ should be interpreted with caution, as the adjusted and unadjusted estimates are not derived from identical samples. Confounder-adjusted models require complete data on all included covariates (age, ethnicity, education, income, and BMI), resulting in the exclusion of participants with missing covariate data. For serum B_12_, this reduced the sample from 959 to 822 omnivores and from 41 to 34 vegetarians. Comparable case loss occurred for the other biomarkers (Table 3). This sample restriction, rather than the covariate adjustment itself, may account for some of the shift between unadjusted and adjusted group means, and the two estimates should not be interpreted as directly comparable measures of the same association.

Serum B_12_ did not differ significantly between omnivores and vegetarians in the present study. Gilsing et al. (2010) reported significantly lower serum B_12_ in vegetarian men compared with omnivores in the EPIC-Oxford cohort (182 vs. 281 pmol/L) [27], and with Herrmann et al. (2003), found low HoloTC in 77% of unsupplemented vegetarians compared with 11% of omnivores, with hyperhomocysteinemia present in 38% of vegetarians versus 16% of omnivores [28]. Consistent with the significant HoloTC and homocysteine differences observed in the present study. Together with broader international evidence documenting consistently lower B_12_ biomarker concentrations in those following plant-based diets without adequate supplementation [10] these findings suggest the pattern observed in UK vegetarian WCBA is not unique to this population but reflects a wider nutritional risk associated with plant-based dietary patterns.

In contrast, folate biomarkers (serum folate and red cell folate) did not differ significantly between omnivores and vegetarians in the cross-sectional comparison, either before or after adjustment. This is a notable finding given that dietary folate intake was significantly higher among vegetarians, suggesting this difference in intake did not translate into a measurable difference in circulating folate status in this sample. Separately, temporal trend analysis (Supplementary Table 2) found that RBC folate declined significantly among omnivores across the study period (*p* < 0.001), while no significant change was observed among vegetarians (*p* = 0.29).However, given the small sample size for the vegetarian group and correspondingly wide confidence intervals, this apparent stability may reflect limited statistical power rather than a genuine protective effect of plant-based diets, and should not be interpreted as evidence against the population-wide decline in folate status reported by Jones et al. [12] without further investigation in a larger sample.

Collectively, these findings indicate a clear nutritional divergence between B_12_ and folate status across dietary patterns, with important implications for one-carbon metabolism and reproductive health [29]. While plant-based diets are beneficial for folate intake and biomarkers, particular attention to vitamin B_12_ intake may be required among individuals following plant-based dietary patterns. Women following plant-based diets may benefit from consistent use of GMP (Good Manufacture Process)-certified B_12_ supplements, evidence indicates that daily doses of 4–20 µg or weekly doses of ≥ 1000 µg are effective at maintaining adequate biomarker status [8, 30].

### Impacts of low B_12_ status during pregnancy

Although this study did not include pregnant participants, these findings have direct relevance to preconception health, as adequate maternal B_12_ status before conception is important for optimal pregnancy and infant outcomes [30]. The forthcoming UK folic acid fortification policy represents an important step toward improving population folate status, and future NDNS monitoring will be crucial to assess its impact across dietary groups, particularly given the metabolic interrelationship between folate and B_12_ [31, 32].

### Implications for dietary reference values for vitamin B_12_

The high prevalence of suboptimal B_12_ biomarker status observed in this study, even among women meeting the current UK RNI of 1.5 µg/day [18], raises important questions about the adequacy of current dietary reference values. Evidence from metabolic and dose-response studies suggests that serum B_12_ and HoloTC plateau only at intakes of approximately 4–7 µg/day, substantially higher than the current UK recommendation [33, 34]. This was particularly evident among vegetarians, where low HoloTC and elevated homocysteine remained common even among those reporting supplement use, though supplement dose, duration, and the direction of this relationship could not be established from cross-sectional data. This pattern is consistent with, rather than direct evidence for, the broader dose-response literature suggesting that current reference values may be insufficient to optimise functional B_12_ status.

The UK RNI for vitamin B_12_, last revised in 1996, was based primarily on preventing megaloblastic anaemia rather than optimising biochemical status [18]. It is now well established that metabolic deficiency can occur in the absence of anaemia [4], and the evidence from our cohort, supported by broader UK data, suggests that relying on haematological criteria underestimates population need. In light of these findings, a revision of UK B_12_ reference intakes should be considered, aligning more closely with the EFSA AI of 4 µg/day, which better reflects requirements for maintaining normal B_12_-dependent metabolic function. Updating UK guidance to recommend intakes ≥ 4 µg/day, particularly for women of childbearing age and those following vegetarian diets, would represent a science-based, preventative approach to reducing the substantial burden of low B_12_ status in the UK.

### Strengths and limitations of the current study

This study offers a comprehensive analysis of vitamin B_12_ and folate intake and biomarker status among WCBA with different diets, using 11 years of nationally representative NDNS data. Its major strengths include the assessment of temporal trends across survey years and the use of validated biomarkers, enhancing the reliability of findings across dietary groups.

However, the division of data into specific time periods (2008–2010, 2010–2012, 2012–2016, and 2016–2019) allowed for trend analysis, but the absence of HoloTC measurements in the earliest period (2008–2010) complicates a comprehensive trend assessment. A limitation is that riboflavin and pyridoxine, which affect homocysteine metabolism, were not analysed, and residual confounding from unmeasured factors such as supplement habits, medication, or genetic variation in folate and B_12_ metabolism cannot be excluded. Dietary intake was based on self-reported food diaries and may therefore be subject to reporting bias or misreporting. In addition, the cross-sectional design prevents causal conclusions.

Because NDNS uses repeated cross-sectional survey samples rather than longitudinal follow-up of individuals, observed changes across survey years represent population-level differences rather than within-person changes over time. While the regression models identified several statistically significant associations with vitamin B_12_ and folate status, their overall explanatory power was modest, with R^2^ values ranging from 0.071 to 0.098 across the four biomarkers (Table 4). This indicates that most of the variance in biomarker concentrations remains unexplained by the measured variables, and the models should not be interpreted as providing strong predictive capacity for individual biomarker status. We should also highlight that the scope of the analysis was limited by the variables available in NDNS dataset; supplement frequency, duration of dietary pattern, and data on genetic variability such as single nucleotide polymorphisms (SNPs) were unavailable and should be explored in future research to enhance model accuracy and interpretation.

### Future research directions and recommendations

From a public health perspective, these findings highlight the need for targeted guidance on vitamin B_12_ status among vegetarian women of childbearing age. Consistent supplementation or consumption of fortified foods is particularly important given the high prevalence of suboptimal biomarker status observed even among those who supplement. Future research should examine whether routine screening of HoloTC and homocysteine in vegetarian women of reproductive age could facilitate earlier identification of those at risk and inform more targeted supplementation guidance.

Finally, ongoing surveillance of the impact of the upcoming UK folic acid fortification policies on folate and B_12_ biomarkers is necessary [31]. Given the metabolic interrelationship between folate and vitamin B_12_, monitoring population status is important to assess whether high folate status in the presence of low vitamin B_12_ status may influence health outcomes, as has been reported in countries with long-standing folic acid fortification programmes [35]. Future NDNS rolling programmes could provide valuable data to evaluate these relationships and inform public health policy.

## Conclusion

This study offers the most comprehensive analysis to date of vitamin B_12_ and folate intake and biomarker status among UK women of childbearing age using 11 years of NDNS data. Omnivores achieved the highest B_12_ intakes and biomarker concentrations. Vegetarians had significantly lower dietary B_12_ intake, consistent with their lower B_12_ status, despite higher rates of B_12_ supplement use, biomarker concentrations remained lower than in omnivores, suggesting that supplementation may not have been sufficient in dose or duration to fully offset lower dietary intake. Folate intake and biomarkers were higher among vegetarians, suggesting a nutritional advantage in this regard. Supplementation was a key determinant of both vitamin B_12_ and folate status across all groups. These findings highlight the need for enhanced awareness and public health messaging around B_12_ supplementation for women following plant-based diets, as well as continued monitoring of folate status in the context of forthcoming national fortification policies.

## Supplementary Information

Below is the link to the electronic supplementary material.

Supplementary Material 1
